# Comparative study of a modified double-lumen tube ventilation control connector and traditional connector in clinical use: a randomised-controlled trial

**DOI:** 10.1186/s12871-022-01816-0

**Published:** 2022-09-06

**Authors:** Chang Liu, Yuanyu Zhao, You Li, Huiwen Guan, Junjie Feng, Shengquan Cheng, Xin Wang, Yue Wang, Xufang Sun

**Affiliations:** Department of Anesthesiology, The Second Hospital of Jilin University, No.218, Ziqiang street, Nanguan District, Changchun City, 130000 Jilin Province China

**Keywords:** double-lumen tube connector, One-lung ventilation, Two-lung ventilation, Bronchial intubation

## Abstract

**Background:**

A Y-shaped rotatable connector (YRC) for double-lumen tubes (DLT) is invented and compared with the traditional connector (Y-shaped connector, YC).

**Methods:**

Sixty patients with ASA grade I-III, aged ≥ 18 years, who needed to insert a DLT for thoracic surgery were recruited and assigned into the YRC group (*n* = 30) and the YC group (*n* = 30) randomly. The primary endpoints included the inhaled air concentration (Fi) and the exhaled air concentration (Et) of sevoflurane before and after the switch between two-lung ventilation and one-lung ventilation at different times, positioning time, and switching time. The secondary endpoints were the internal gas volume of the two connectors, airway pressure, and the sputum suction time.

**Results:**

The Et and Fi of the YRC group and the YC group were significantly different (all *p* < 0.05) at 5s, 10s, and 30s after the patient switched from two-lung ventilation to one-lung ventilation. The positioning time of the YRC group was less than YC group (89.75 ± 14.28 s vs 107.80 ± 14.96 s, *p* < 0.05), as well as the switching time (3.60 ± 1.20 s vs 9.05 ± 2.53 s, *p* < 0.05) and the internal gas volume (17.20 ml vs 24.12 ml). There was no difference in airway pressure and the sputum suction time in two groups.

**Conclusion:**

Compared with YC, YRC was beneficial for maintaining depth of anesthesia, improves efficiency for the switch between one-lung and two-lung ventilation, and shortens the tube positioning time.

## Highlights

1. YRC was beneficial to maintain the stability of anesthesia depth.

2. YRC improves the conversion efficiency for one-lung and two-lung ventilation.

3. YRC shortens the positioning time.

## Background

During thoracic surgery, it is necessary to insert a double-lumen tube (DLT) to achieve lung isolation and one-lung ventilation [1]. After intubation, a connector connecting the DLT to the breathing circuit of the anesthesia ventilator was used to realize the conversion between two-lung and one-lung ventilation when necessary [2]. For example, positioning the tracheal tube by auscultation, fully exposing the pathological changes of the lung surface during the operation, and checking the air leakage of the tracheal stump [3–5]. Usually, one-lung ventilation can be achieved by clamping one side of the DLT with a surgical clamp, but this deforms the tube on the clamped side, which may result in increased resistance to gas flow through the tube when ventilation is restored. After the weight of the surgical clamp and the YC are connected together, the instability of the connector will increase, which will easily lead to the connector falling off. In our department, the traditional Y-shaped connector (YC) coming with DLT is generally used alternately with the threaded pipe connector when the ventilation mode is switched. This ventilation mode has poor conversion efficiency and the patient needs to completely disconnect the breathing path during conversion, resulting in leakage of inhaled anesthetics and unstable of anesthesia depth [6].

To improve the shortcomings of this connector in the ventilation conversion, we invented the Y-shaped rotatable connector (YRC) (authorized by the national utility model patent: CN 208481834 U). The conversion of two ventilation modes can be realized by twisting the Y-type valve outside the connector. However, the clinical performance and outcomes of YRC compared with YC remains to be investigated.

In the study, we designed a randomized controlled trail to compare the clinical performance of the YRC and the traditional YC. We assume that the YRC reduces the leakage of sevoflurane when the ventilation mode is switched, shortens the positioning time of the tracheal tube, and improves the conversion efficiency of the one-lung and two-lung ventilation modes. At the same time, sevoflurane is often used in thoracic surgery because of its low blood/gas distribution coefficient, rapid induction and recovery process, easy control of anesthesia depth, and pulmonary protection [7, 8]. This result may facilitate ventilation management in thoracic surgery.

## Methods

This clinical study has been registered in Chinese Clinical Trial Registration Center (ChiCTR, www.chictr.org.cn, registration ID: ChiCTR2000040188, registration data: 24–11-2020). This study was approved by the ethics committee of the Second Hospital of Jilin University, Changchun, China on November 23, 2020 (the approval number of the ethics committee: 2020–138). Written informed consent was obtained from all patients. All methods were performed in accordance with the relevant guidelines and regulations.

### Patients and groups

This study included 60 patients with the American Society of Anesthesiologists (ASA) classification I to III, aged ≥ 18 years old, and undergoing a DLT placement for thoracic surgery. Exclusion criteria were: patients younger than 18 years of age, emergency thoracic surgery, pregnancy, and who had contraindications for DLT, such as laryngeal edema, acute laryngitis, etc., and patients who were predicted to have a difficult intubation, such as a known airway occupying, Mallampati grade III or IV, severely restricted head and neck movement, etc. [9, 10]. Patients underwent simple randomization into YRC group (Y-shaped rotatable adjustment between triple lumen tube and DLT connector, *n* = 30) and the YC group (the traditional connector after intubation, *n* = 30) according to computer generated random number.

### Primary and secondary endpoints

The tracheal intubation, positioning, conversion from two to one-lung ventilation, and sputum suction of the two groups of patients were completed by 5 full-time thoracic anesthesiologists who had similar working hours and work experience, and were proficient in the use of the two connectors. The same staff recorded the endpoints. The primary endpoints included the inspired (Fi) and expired (Et) sevoflurane concentration, positioning time (from the endotracheal tube inserting into the trachea to that the auscultation method and fiberoptic bronchoscopy were used to locate the endotracheal tube), and time during two-lung and one-lung ventilation that before and after the switch between two-lung and one-lung ventilation in the two groups of patients were recorded. The secondary endpoints were the internal gas volume of the two connectors, airway pressure, and the sputum suction time. The internal gas volume of the two connectors was measured [11], and after DLTs positioning, the peak airway pressure, plateau pressure, and average pressure of the patients at 5 min of two-lung ventilation of the two groups of patients were recorded. The time required to suck sputum from the non-ventilated lung during one-lung ventilation. This time is defined as the time from when the suction tube is connected to the suction device to the end of the suction tube completely leaving the opening of the DLT. The time required for one-lung ventilation to suck sputum from the ventilated lung. This time is defined from the sputum suction tube is connected to the aspirator to the end of the sputum suction and the patient returns to normal one-lung ventilation.

The internal gas volume of the two connectors was measured as follow. Seal the ends of the two connectors connected to the breathing circuit with tape, pour water into them from the end of the connecting tracheal tube until the connector is completely filled with water, and measure the milliliters of the injected water, that is, the internal gas volume value of the two connectors.

### YRC

**Fig. 1:**
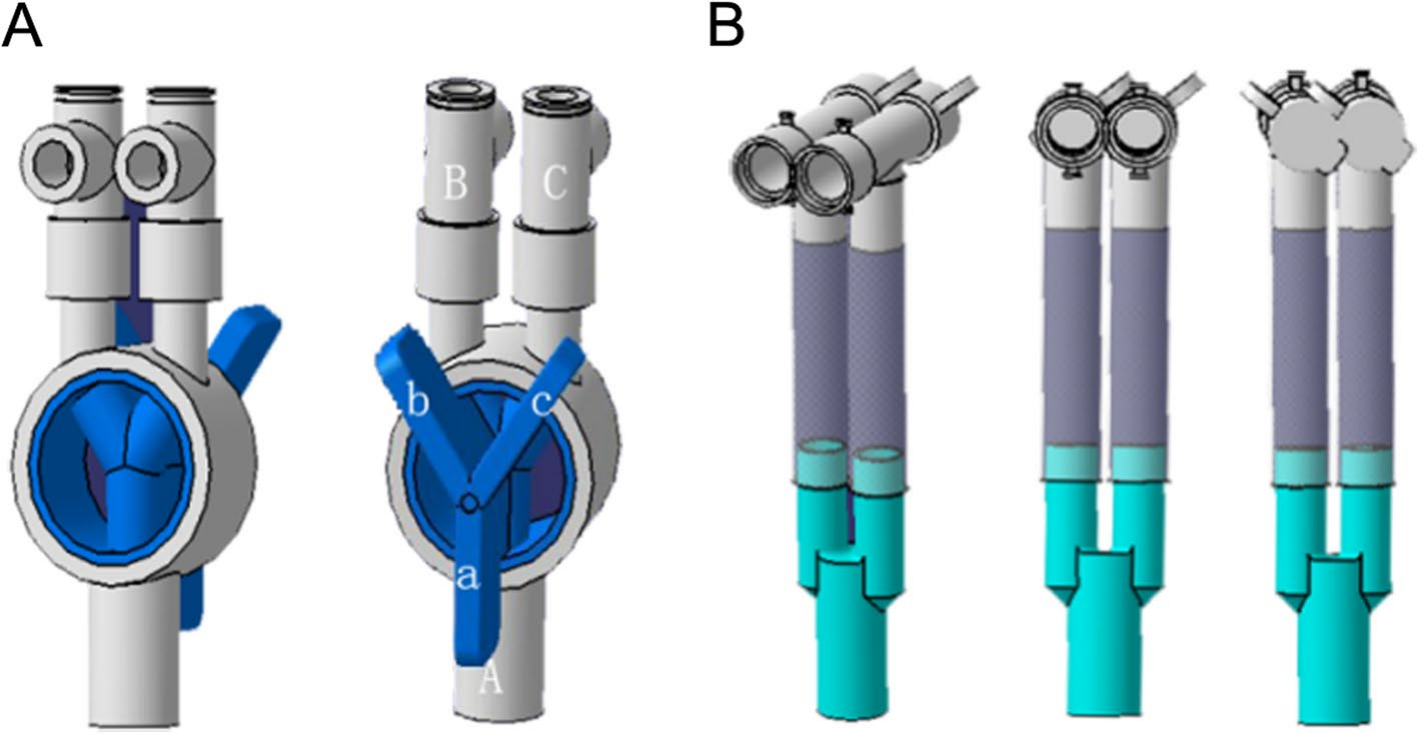
The Y-shaped rotatable connector (YRC, **A**) and the traditional Y-shaped connector (YC, **B**)

The YRC invented by the author of this article (Fig. 1 A) has a Y-type rotatable valve on its surface. Rotating the valve can drive the Y-type interconnected pipes inside, thereby controlling the direction of gas flow in the valve. Y-type valves b and c are at an angle of 90°, and their corresponding internal pipes are also at an angle of 90°. Y-type valves b and c are at an angle of 135° to a, respectively, and their corresponding internal pipes are also at an angle of 135°. After connecting the DLT and breathing circuit with the traditional YC valve (Fig. 1 B), although one-lung ventilation can also be achieved through the surgical clamp, the tube on the clamp side is prone to deformation. The weight of the surgical clamp can easily lead to instability of the connector, and the operation is cumbersome.

**Fig. 2:**
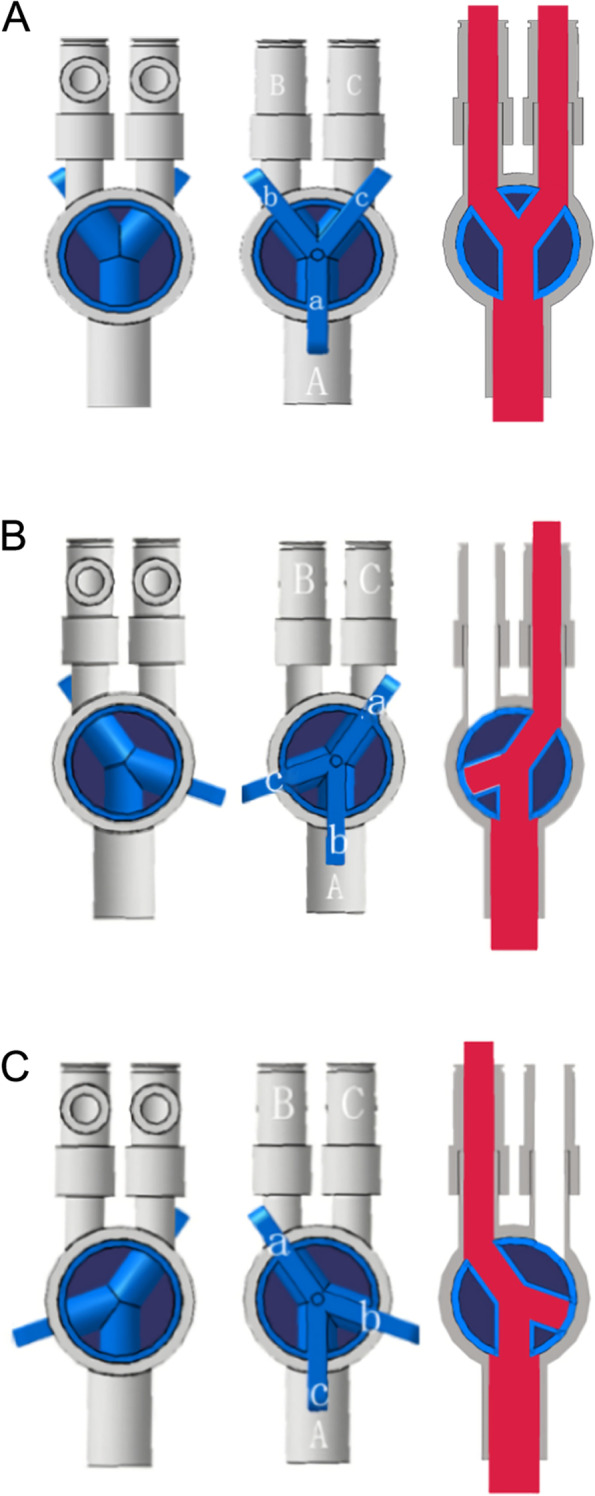
The YRC used for two-lung ventilation (**A**) and one-lung ventilation (**B** and **C**)

**Fig. 3:**
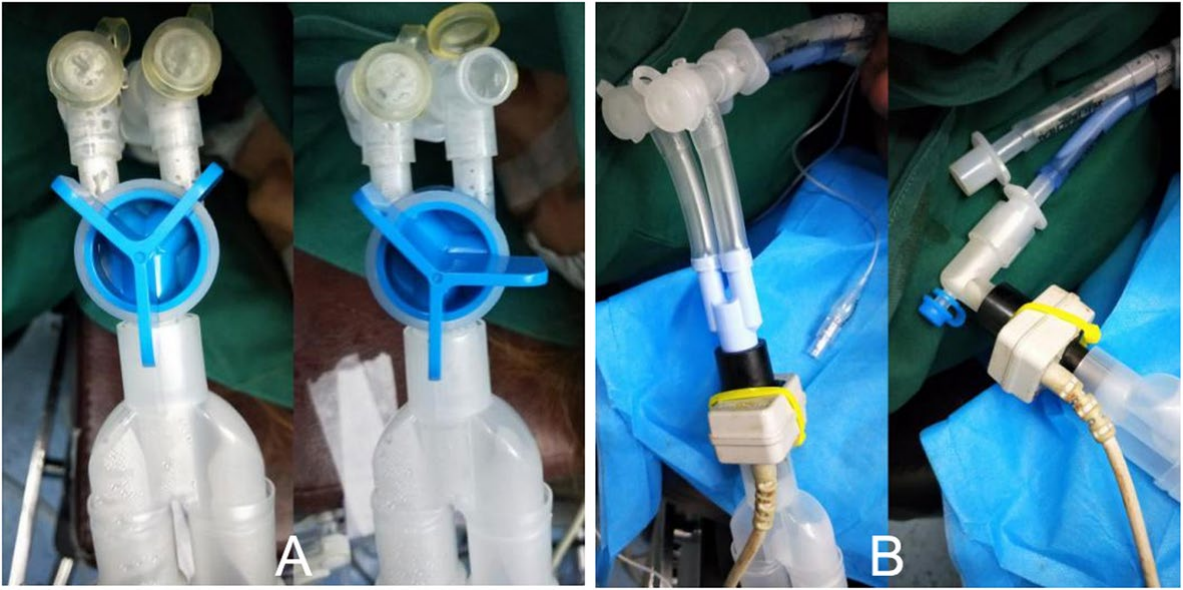
The clinical application of YRC (**A**) and YC (**B**)

When the YRC was used for two-lung ventilation, the b and c ends of the Y-type valve point to the B and C ends that connect the opening of the DLT respectively, and the a end points to the A end that connects to the breathing circuit (Fig. 2 A). When one lung is ventilated, only need to rotate the Y-valve so that the a-end points to the side where the DLT needs to be ventilated (Fig. 2 B). If end a point to end B, end c points to end A, and end b is closed. At this time, the open side of the DLT connected to end B is ventilated, and the open side of the DLT connected to end C is not ventilated. If end a points to end C, end b points to end A, and end c is closed. At this time, the open side of the DLT connected to end C is ventilated, and the open side of the DLT connected to end B is not ventilated (Fig. 2C). It can be seen that it can be achieved by turning the Y-valve when switching between one-lung and two-lung ventilation modes. The clinical application was shown in Fig. 3.

### Comparative study

All patients were fasting before the operation, intravenous access was established, and routinely monitored by the electrocardiogram, invasive arterial blood pressure, pulse oximetry, and bispectral index (BIS). The appropriate DLT size and DLT type (left-sided or right-sided) were selected according to the patient's preoperative CT scan. Lidocaine cream was applied to the cuff at the front of the tube to provide lubrication and surface anesthesia. During the induction of general anesthesia, penehyclidine (1 mg), midazolam (0.05 mg/kg), propofol (2 mg/kg), sufentanil (0.5ug/kg), cis-atraline Curonium (0.2 mg/kg) were injected intravenously. In the induction process, the patient was ventilated with a pure oxygen mask for 3 min, and 2% lidocaine was injected into the patient’s larynx and trachea 1 min before tracheal intubation for topical anesthesia, and then tracheal intubation was performed. The anesthesiologist used a UEScope® video laryngoscope (TDC-C3) to expose the glottis to make the blue cuff at the bronchial end of the DLT completely passed through the glottis, pulled out the stylet of the DLT, and then rotated the DLT to the bronchus according to the type of DLT, push it towards the bronchus until moderate resistance is felt, and continue to inflte the two cuffs. Then the YRC group was connected to the YRC to switch between one-lung and two-lung ventilation by rotating the Y-valve, and the YC group was switched between one-lung and two-lung ventilation by alternately using YC and threaded tube connectors. The DLT of both groups were adjusted to a satisfactory position, that is, clear breath sounds in both lungs during dual-lung ventilation, and clear breath sounds on the ventilated side during one-lung ventilation (especially in the upper right lobe), no breath sounds on the non-ventilated side. Fiberoptic bronchoscopy is intuitive and reliable in DLT localization and inspection, and is the gold standard for rapid and accurate determination of DLT location. Then, the fiberoptic bronchoscope was used to determine the position of the DLT, the tube was fixed and connected to a ventilator at the same time for mechanical ventilation of both lungs. The tidal volume was 7 ml/kg, the frequency was 14 beats/min, the PEEP was 8, and the end-tidal carbon dioxide partial pressure is maintained at 35 ~ 45mmHgg (1 mmHg = 0.133 kPa). After ventilation of both lungs, patients received sevoflurane inhalation (3% vol) and oxygen flow rate of 2 l/min. During the operation, remifentanil (0.2 ug/kg/min) was continuously applied intravenously, and cis-atracuramide was injected intermittently to maintain muscle relaxation. After changing the position, the fiberoptic bronchoscope was used to adjust the position of the tube, and switch to one-lung ventilation before pleura was opened [12, 13].

### Sample size calculation

Using G.Power3.1 software, the required sample size of each group was 29 according to the mean and standard deviation in the pre-experiment with the following settings: test family = t-tests (two-tailed); statistical tests = difference of means between two independent groups; effect size d computed from means and standard deviations of groups to compare; α error probability = 0.05; power (1–β error probability) = 0.08 (It is generally believed that a statistical power of 0.8 is an acceptable threshold value). Power analysis was performed according to the primary endpoint (switching time). However, in order to prevent insufficient sample size resulted by various reasons, preliminary qualification assessments were performed on 64 patients, and finally 60 patients participated in the analysis of this study (30 patients per group).

### Statistical analysis

The data was analyzed by SPSS25.0 statistical software. Measurement data conforming to the normal distribution were expressed as mean ± standard deviation (x ± s). Independent sample t test was used for comparison between groups, and x^2^ test was used for count data. *P* < 0.05 means that the difference is statistically significant.

## Result

### The general data of patients in two groups

A total of 64 patients were evaluated for preliminary eligibility. Among them, 3 patients refused to sign the study consent form, 1 case was excluded due to a difficult intubation after preoperative evaluation, and finally 60 patients participated in the study analysis, including 44 cases of lung space‐occupying lesions, 2 cases of pneumothorax, and 5 cases of mediastinal tumor, 3 cases of esophageal lesions, 3 cases of empyema, 1 case of pericardial effusion, 1 case of rib malignant tumor, and 1 case of rib fracture. The biometric data of the two groups of patients were not statistically significant (Table 1).

**Table 1:** The general characteristics of patients

Variable	YRC group (*n* = 30)	YC group (*n* = 30)	*P* value
Gender (male/females)	15/15	14/16	0.8
Age (years)	58.7 ± 11.8	56.5 ± 10.5	0.712
Height (cm)	166.3 ± 8.9	165.9 ± 8.2	0.836
Weight (kg)	65.5 ± 12.4	63.9 ± 11.2	0.633
ASA (I/II)	1/29	2/28	0.561
DLT type (left/right)	22/8	20/8	0.581
DLT size			
Fr35	10	8	/
Fr37	15	16	/
Fr39	5	6	/
Type of surgery			
Lobectomy of lungs	8	10	/
Pulmonary wedge resection	13	12	/
Pneumothorax	/	2	/
One-side pneumonectomy	1	/	/
Mediastinal tumor	3	2	/
Esophageal lesions	1	2	/
Empyema	3	/	/
Pericardial effusion	/	1	/
Rib malignant tumor	/	1	/
Rib fracture	1	/	/

*ASA* American Society of Anesthesiologists, *DLT* Double-lumen tubes

### The Fi and Et sevoflurane concentration were different during two-lung and one-lung ventilation in two groups

**Fig. 4:**
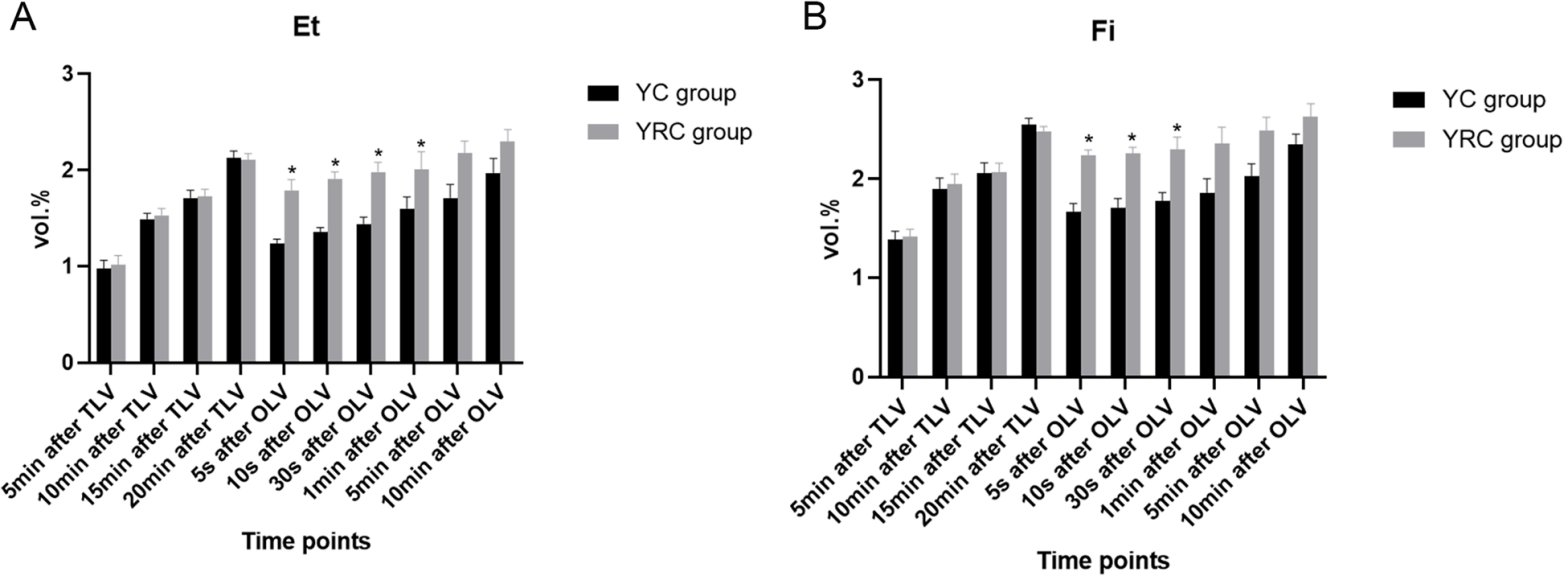
The inspired (Fi) and expired (Et) sevoflurane concentration during two-lung and one-lung ventilation in two groups. * Compared with the YC group, *P* < 0.05. TLV: two-lung ventilation, OLV: one-lung ventilation

**Fig. 5:**
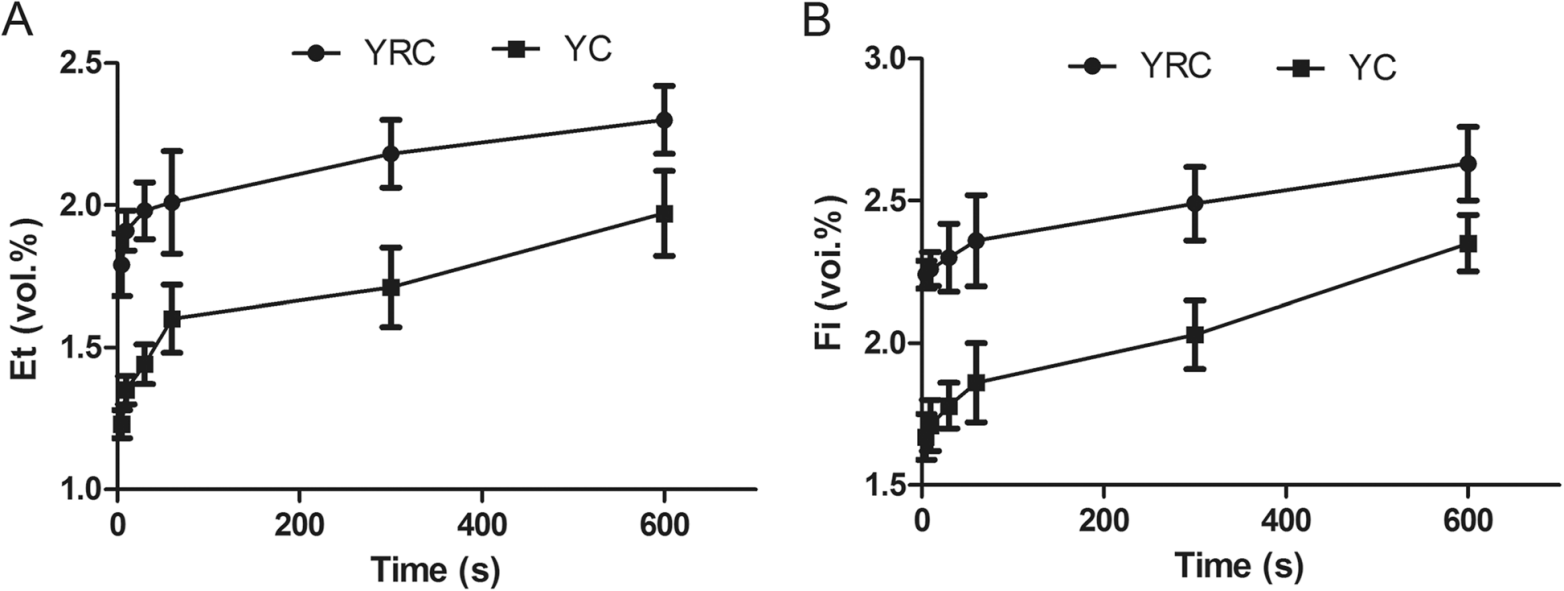
The inspired (Fi) and expired (Et) sevoflurane concentration during one-lung ventilation in two groups

The data showed that the Et and Fi of the YRC group and the YC group were significantly different (all *p* < 0.05) at 5 s, 10 s, and 30 s after the patient switched from two-lung ventilation to one-lung ventilation (Table 2, Fig. 4). The shift of Et and Fi of YRC were smaller than those of YC, and the concentration of inhaled anesthetics changes smaller (Fig. 5).

**Table 2:** The inspired (Fi) and expired (Et) sevoflurane concentration during double-lung and single-lung ventilation in two groups

Time points	Et (vol.%)			Fi (vol.%)		
	YRC group (*n* = 30)	YC group (*n* = 30)	*P* value	YRC group (*n* = 30)	YC group (*n* = 30)	*P* value
5 min after double-lung ventilation	1.01 ± 0.10	0.97 ± 0.09	0.284	1.41 ± 0.08	1.38 ± 0.09	0.261
10 min after double-lung ventilation	1.53 ± 0.07	1.49 ± 0.06	0.202	1.95 ± 0.10	1.90 ± 0.11	0.302
15 min after double-lung ventilation	1.73 ± 0.07	1.71 ± 0.08	0.235	2.07 ± 0.09	2.06 ± 0.10	0.303
20 min after double-lung ventilation	2.11 ± 0.06	2.13 ± 0.07	0.201	2.48 ± 0.05	2.55 ± 0.06	0.284
5 s after single-lung ventilation	1.79 ± 0.11^a^	1.23 ± 0.05^a^	0.004	2.24 ± 0.05^a^	1.67 ± 0.08^a^	0.006
10 s after single-lung ventilation	1.91 ± 0.07^a^	1.35 ± 0.05^a^	0.035	2.26 ± 0.06^a^	1.71 ± 0.09^a^	0.015
30 s after single-lung ventilation	1.98 ± 0.10^a^	1.44 ± 0.07^a^	0.028	2.30 ± 0.12^a^	1.78 ± 0.08^a^	0.014
1 min after single-lung ventilation	2.01 ± 0.18^a^	1.60 ± 0.12^a^	0.015	2.36 ± 0.16	1.86 ± 0.14	0.236
5 min after single-lung ventilation	2.18 ± 0.12	1.71 ± 0.14	0.211	2.49 ± 0.13	2.03 ± 0.12	0.332
10 min after single-lung ventilation	2.30 ± 0.12	1.97 ± 0.15	0.114	2.63 ± 0.13	2.35 ± 0.10	0.078

^a^ Statistically significant

### The conversion efficiency was improved in YRC

Compared with YC, YRC can improve the conversion efficiency of DLTs for one-lung and two-lung ventilation, and reduce the positioning time, but there is no difference on the sputum suction operation (Table 3). The positioning time of the YRC group was shorter than that of the YC group (89.75 ± 14.28 s vs 107.80 ± 14.96 s, *p* < 0.01) (Table 3). The switching time of the YRC group was shorter than that of the YC group (3.60 ± 1.20 s vs 9.05 ± 2.53 s, *p* < 0.01) (Table 3). The sputum suction time of the YRC group was not statistically different from that of the YC group (8.79 ± 1.95 s vs 9.64 ± 1.77 s, *p* = 0.081, 13.56 ± 1.81 s vs 14.11 ± 1.68 s, *p* = 0.304) (Table 3).

**Table 3:** The observation indicators of two groups

Variable	YRC group (*n* = 30)	YC group (*n* = 30)	*P* value
Intubation time (s)	89.75 ± 14.28	107.80 ± 14.96	0.00^b^
Switching time (s)	3.60 ± 1.20	9.05 ± 2.53	0.00^b^
Sputum suction time for non-ventilate side lung (s)	8.79 ± 1.95	9.64 ± 1.77	0.081
Sputum suction time for ventilate side lung (s)	13.56 ± 1.81	14.11 ± 1.68	0.304
Internal gas volume of connectors (ml)	17.20	24.12	0.500

^b^ Statistically significant

### The mechanical dead space was reduce in YRC

The internal gas volume of the two connectors was measured. The results are averaged for three times, showing that the internal gas volume of YRC was 17.20 ml and the internal gas volume of YC was 24.12 ml (Table 3). This result showed that YRC can reduce the mechanical dead space in the breathing circuit.

### The airway pressure during two-lung and one-lung ventilation in two groups was not significantly different

There was no difference in the peak airway pressure, plateau pressure, and average pressure of patients with bilateral lung ventilation for 5 min, nor in the peak airway pressure, plateau pressure, and average pressure of patients with one-lung ventilation for 5 min in two groups (*P* > 0.05) (Table 4). This result showed that YRC can achieve the tightness and safety of overall airway during mechanical ventilation.

**Table 4:** The airway pressure during double-lung and single-lung ventilation in two groups

Groups	Double-lung ventilation			Single-lung ventilation		
	Ppeak (cmH_2_O)	Pplat (cmH_2_O)	Pmean (cmH_2_O)	Ppeak (cmH_2_O)	Pplat (cmH_2_O)	Pmean (cmH_2_O)
YRC group (*n* = 30)	16.05 ± 2.19	14.10 ± 1.87	6.21 ± 0.79	24.68 ± 3.29	21.50 ± 2.65	9.90 ± 1.22
YC group (*n* = 30)	15.95 ± 2.34	13.79 ± 1.55	5.92 ± 0.71	23.96 ± 2.60	20.98 ± 2.02	9.26 ± 0.98
*P* value	0.380	0.104	0.310	0.206	0.094	0.078

## Discussion

In thoracic surgery, a DLT is required to separate the two sides of the thoracic cavity [14]. The traditional YC connecting the DLT and the respiratory circuit can be achieved by alternating with threaded fittings/using a surgical clamp to seal the side of the DLT in the conversion of one-lung and two-lung ventilation. The use of the surgical clamp easily deforms the tube on the clamped side, which increases the resistance to gas flow in the tube after ventilation is resumed [15]. After the weight of the surgical clamp is integrated with the YC, the instability of the connector is increased, which is easy to cause the connector to fall off. The deficiency of using DLT interchangeably with threaded fitting is that the operation of ventilation conversion process is complicated, and the repeatedly inserting and pulling may cause the shedding or loss of the connector. If the shedding of connector is not detected in time, it will affect the tightness of the whole respiratory circuit during anesthesia, cause hypoxia in patients, and even threaten the safety of patient's life [16]. If the connector is lost, the anesthesiologist's fluency in ventilation switch is reduced and the operation process is delayed. One-lung ventilation differs from two-lung ventilation in terms of pulmonary respiratory dynamics, ventilation and ventilation function, especially pulmonary blood flow [17]. During one-lung ventilation, the uptake of inhaled anesthetics in the lungs at the same inhaled concentration is less than that in two-lung ventilation, and the concentration of anesthetics in exhaled air is higher. Therefore, compared with two-lung ventilation, the depth of anesthesia in one-lung ventilation is shallower at the same time point, and the time required for inhalation anesthesia to reach steady state is longer [18]. Traditionally, potent inhalation anesthetics have been the drugs of choice for one-lung ventilation because they reduce airway responsiveness by acting directly on the airway smooth muscle tissue of the bronchi [19]. However, the traditional connector needs to completely disconnect the respiratory circuit during the conversion of one-lung ventilation and two lung ventilation, and the conversion time is relatively long, which will not only make a large number of inhaled anesthetics leak from the respiratory circuit and pollute the air in the operating room, but also greatly affect the stability of the anesthesia depth of patients.

To solve the above problems, we invented YRC which dispense with disconnect the respiratory circuit completely during the conversion of one-lung and two-lung ventilation. It can realize the conversion of ventilation mode only by rotating the Y-type valve easily and fastly. Et is approximated as alveolar gas concentration, which can provide reliable data support for inhalation anesthesia. From the results of this study, it can be seen that there were significant differences in Et and Fi between YRC group and YC group at 5 s, 10 s and 30 s after the conversion of two-lung ventilation to one-lung ventilation. The shift of Et and Fi of YRC were smaller than those of YC, and the concentration of inhaled anesthetics changes smaller, which benefits to stability and control of the anesthetic state [20, 21]. In addition, the anesthesiologists need to frequently convert the ventilation mode when they use the auscultation method to locate the endotracheal tube after tracheal intubation. In addition, compared with YC, YRC greatly reduces the conversion time of one-lung and two-lung ventilation of the DLT, thereby reducing the positioning time, and reduces the non-ventilation time of the patients. For patients with relatively poor lung function and poor oxygen storage, such as chronic obstructive pulmonary diseases, pulmonary interstitial fibrosis and other diseases, it is crucial to shorten the time of non-ventilation during intubation [6, 22], which improves the safety of patients during intubation. Moreover, the internal gas volume of YRC was smaller than that of YC, and YRC could reduce the mechanical dead space in the respiratory circuit, thereby reducing the mechanical compliance and ventilation loss, and improving the ventilation efficiency [23].

The Y-type rotatable valve core is the key value of the invention. It not only realizes the free conversion of one-lung and two-lung ventilation, but also has the advantages of saving time and labor, low cost and disposable use in operation. It is no different from the traditional Y connector in airway tightness and safety, which can also ensure the health and safety of patients’ respiratory system.

However, there are still some limitations. First of all, all the anesthesiologists involved in the study were not blinded to the connectors used, and there may be some observer bias. However, it is difficult to solve this problem in the comparative study of the application of the two connectors in thoracic surgery, and the recorded data such as time are completed by the same staff who does not understand the experiment, and the recorded endpoints are objective. Another limitation is that the positioning time of different anesthesiologists is a relatively subjective factor, but we chose five anesthesiologists with similar working time and working experience to reduce the error caused by this factor. At the same time, each of them not only participated in the intubation of the YRC group but also participated in the intubation of the YC group. Finally, due to the small sample size of this study, more trials with larger sample sizes are needed for validation.

## Conclusion

In conclusion, Compared with YC, YRC was beneficial for maintaining depth of anesthesia, improves efficiency for the switch between one-lung and two-lung ventilation, and shortens the tube positioning time. Therefore, YRC has certain practicability in the clinical application.

## Data Availability

All data generated or analysed during this study are included in this published article.

## Abbreviations

YC: Y-shaped connector
BIS: Bispectral index
DLT: Double-lumen tube
ASA: American Society of Anesthesiologists
BIS: Bispectral Index
